# Activation of *gab* cluster transcription in *Bacillus thuringiensis* by γ-aminobutyric acid or succinic semialdehyde is mediated by the Sigma 54-dependent transcriptional activator GabR

**DOI:** 10.1186/s12866-014-0306-3

**Published:** 2014-12-20

**Authors:** Qi Peng, Min Yang, Wei Wang, Lili Han, Guannan Wang, Pengyue Wang, Jie Zhang, Fuping Song

**Affiliations:** State Key Laboratory for Biology of Plant Diseases and Insect Pests, Institute of Plant Protection, Chinese Academy of Agricultural Sciences, Beijing, China; College of Life Sciences, Northeast Agriculture University, Harbin, China

**Keywords:** GabR, Sigma 54, GABA, SSA, PAS domain

## Abstract

**Background:**

*Bacillus thuringiensis* GabR is a Sigma 54-dependent transcriptional activator containing three typical domains, an N-terminal regulatory domain Per-ARNT-Sim (PAS), a central AAA^+^ (ATPases associated with different cellular activities) domain and a C-terminal helix-turn-helix (HTH) DNA binding domain. GabR positively regulates the expression of the *gabT* gene of the *gab* gene cluster, which is responsible for the γ-aminobutyric acid (GABA) shunt.

**Results:**

Purified GabR was shown to specifically bind to a repeat region that mapped 58 bp upstream of the *gabT* start codon. The specific signal factors GABA and succinic semialdehyde (SSA) activated *gabT* expression, whereas GABA- and SSA-inducible *gabT* transcription was abolished in *sigL* and *gabR* mutants. GABA and SSA did not induce the expression of either SigL or GabR. Deletion of the PAS domain of GabR resulted in increased *gabT* transcriptional activity, both in the presence and absence of GABA.

**Conclusions:**

This study identified the GabR-binding site on the *gabT* promoter; however, GabR does not bind to its own promoter. *gabT* transcription is induced by GABA and SSA, and inducible expression is dependent on SigL and activated by GabR. The PAS domain in GabR is repressing its enhancer transcriptional activity on the *gabT* promoter. Repression is released upon GABA addition, whereupon transcription is induced.

**Electronic supplementary material:**

The online version of this article (doi:10.1186/s12866-014-0306-3) contains supplementary material, which is available to authorized users.

## Background

The non-coded amino acid γ-aminobutyric acid (GABA) is found in a wide range of organisms, including bacteria, yeasts, plants and animals. In the adult brain, it is the predominant inhibitory neurotransmitter [1]. The GABA shunt, in which glutamate is converted to succinate, with GABA as an intermediate, is ubiquitous in most prokaryotic and eukaryotic organisms. Two important enzymes are involved in the GABA shunt: GABA transaminase (GABA-T, EC 2.6.1.19, encoded by *gabT*), which catalyzes the reversible conversion of GABA to succinic semialdehyde (SSA), and succinic semialdehyde dehydrogenase (SSADH, EC 1.2.1.16, encoded by *gabD*), which irreversibly oxidizes SSA to succinate [2]. Both *gabT* and *gabD* have been identified in *Escherichia coli* [3], *Rhizobium leguminosarum* [4] and *Bacillus subtilis* [5].

*Bacillus thuringiensis* (Bt) has been widely commercialized throughout the world because of the production by strains of this species of insecticidal crystal proteins. During sporulation, Bt produces large crystalline parasporal inclusions. Previous studies suggested that the GABA shunt was related to the formation of Bt crystals and spores [6]. Bt possess a modified tricarboxylic acid cycle (TCA) in which α-ketoglutarate dehydrogenase is absent. Instead, α-ketoglutarate is converted to glutamate and then to succinate via γ-aminobutyric acid [7]. The GABA shunt allows circumvention of the α-ketoglutarate dehydrogenase step. Enzymatic analyses of Bt extracts suggested that a modified TCA operates during the sporulation phase of growth in conjunction with the glyoxylic acid cycle and the GABA pathway [6]. The Bt *gab* gene cluster is involved in GABA degradation. Previously, we proposed a model to explain the expression and regulation of the *gab* gene cluster in Bt strain HD73 [8]. In this model, three genes (*gabT*, *gabR* and *gabD*) form two transcriptional units. *gabT* is separately transcribed, while *gabR* and *gabD* are co-transcribed. Moreover, we found that Sigma 54 RNA polymerase holoenzyme transcribes *gabT*, with transcription activated by GabR.

Bacterial enhancer binding proteins (bEBPs) are transcriptional activators that assemble as hexameric rings in their active form, using ATP hydrolysis to modify the conformation of RNA polymerase containing the alternative sigma factor Sigma 54 [9]. bEBPs generally consist of three domains: the N-terminal regulatory domain plays a role in signal perception and modulates the activity of bEBP; the central AAA^+^ domain is responsible for nucleotide binding, ATP hydrolysis, and oligomerization, and is sufficient to activate Sigma 54-dependent transcription; and the C-terminal DNA-binding domain contains a helix-turn-helix (HTH) motif that enables enhancer site recognition by specific upstream activating sequences (UAS) [9]. The N-terminal or regulatory domains of many bEBPs respond to various environmental signals and thereby regulate the activity of the central AAA^+^ domain [9]. For example, NtrC, DctD, ZraR and FlgR bEBPs are part of two-component systems that couple an external stimulus to an internal response [10-13]. These bEBPs have response regulator domains that are phosphorylated by specific sensor kinases. Certain other Sigma 54 activators have a regulatory domain that binds to small effector molecules, such as FhlA, which contains two GAF domains that bind formate to activate transcription of the formate hydrogen lyase system [14]. PAS (Per-ARNT-Sim) domains have been shown to bind a diverse array of ligands, including heme, flavins, 4-hydroxycinnamic acid, carboxylic acids, and divalent metal ions [15]. However, very few of the signals sensed by PAS domains in bEBPs have been identified. This is the case of TyrR bEBP-like protein, which in the presence of phenylalanine, tyrosine, or tryptophan, proposed to be sensed by the PAS domain, interact with the alpha-subunit of RNA polymerase to activate transcription [16].

GabR is a Sigma 54-dependent transcriptional activator that has three typical domains: an N-terminal PAS regulatory domain, a central AAA^+^ domain, and a C-terminal HTH DNA-binding domain. In this study, we demonstrated that GabR binds upstream and in close proximity to the Sigma 54 binding site in the *gabT* promoter. Our data also show that the PAS domain in GabR is repressing its enhancer transcriptional activity on the *gabT* promoter. Repression is released upon GABA addition, whereupon transcription is induced.

## Methods

### Bacterial strains, plasmids, and growth condition

The bacterial strains and plasmids used in this study are listed in Table 1. Bt strain HD73 was used throughout the study (accession numbers CP004069) [17]. *E. coli* strain TG1 was used as the host for cloning experiments. The Dam^−^/Dcm^−^*E. coli* ET12567 strain (laboratory stock) was used to generate unmethylated DNA for the electrotransformation assay. Bt strains were transformed by electroporation, as described previously [18,19]. *E. coli* and *Bt* strains were cultured in Luria-Bertani (LB) medium, with 220 rpm shaking, at 37 and 30°C, respectively. The antibiotic concentrations used for bacterial selection were as follows: 100 μg/ml kanamycin, 10 μg/ml erythromycin, and 10 μg/ml tetracycline for Bt, and 100 μg/ml ampicillin for *E. coli*. For β-galactosidase assays, bacteria were grown on Association of Official Analytical Chemists (AOAC) medium (Sigma, S5431, Saint-Quentin Fallavier, France) containing glucose (7 mM) and Schaeffer’s sporulation medium (SSM) [20]. Transcription from the Bt *gabT* promoter was induced by adding 0, 1, 2, or 5 mM of SSA and GABA to the AOAC culture medium.

**Table 1:** **Strains and plasmids used in this study**

**Strain or plasmid**	**Relevant genotype and characteristics**	**Reference or source**
**Strains**		
HD73	Bt subsp. *Kurstaki* carrying the *cry1Ac* gene	Laboratory collection
HD(P*gabT*-*lacZ*)	Bt HD73 carrying pHT-PgabT plasmid; Em^R^	[8]
HD(P*gabR*-*lacZ*)	Bt HD73 carrying pHT-PgabR plasmid; Em^R^	[8]
HD(P*gabTDR-lacZ*)	Bt HD73 carrying pHT-PgabTDR plasmid; Em^R^	This study
HD(P*gabTDI-lacZ*)	Bt HD73 carrying pHT-PgabTDI plasmid; Em^R^	This study
HD(P*sigL*-*lacZ*)	Bt HD73 carrying pHT-PsigL plasmid; Em^R^	This study
Δ*gabT*(P*gabT*-*lacZ*)	HD(Δ*gabT*) carrying pHT-PgabT plasmid; Em^R^	[8]
Δ*gabD*(P*gabT*-*lacZ*)	HD(Δ*gabD*) carrying pHT-PgabT plasmid; Em^R^	[8]
Δ*gabR*(P*gabT*-*lacZ*)	HD(Δ*gabR*) carrying pHT-PgabT plasmid; Em^R^	[8]
Δ*sigL*(P*gabT*-*lacZ*)	HD(Δ*sigL*) carrying pHT-PgabT plasmid; Em^R^	[8]
DPAS(P*gabT*)	HD(Δ*gabR*) carrying pHT-PgabT and pHT1618-DPAS plasmid; Em^R^ and Tet^R^	This study
C*gabR*(P*gabT*)	HD(Δ*gabR*) carrying pHT-PgabT and pHT1618-gabR plasmid; Em^R^ and Tet^R^	This study
*E. coli* TG1	Δ(*lac-proAB*) *supE thi hsd-*5 (*F’ traD36 proA* ^+^ *proB* ^+^ *lacI* ^q^ *lacZ*ΔM15), general purpose cloning host	Laboratory collection
*E. coli* ET12567	*F* ^−^ *dam-13*::Tn*9 dcm-6 hsdM hsdR recF143 zjj-202*::Tn*10 galK2 galT22 ara14 pacY1 xyl-5 leuB6 thi-1*, for generation of unmethylated DNA	Laboratory collection
*E.coli* BL21(DE3)	*E.coli B, F* ^*−*^ *, dcm, ompT, hsdS(rB-mB-), gal, λ(DE3)*	Laboratory collection
BL21 (pET21b-*gabR*)	BL21(DE3) with pET21b-gabR plasmid	This study
**Plasmids**		
pET21b	Expressional vector, Ap^R^, 5.4 kb	Laboratory collection
pHT304-18Z	Promoterless *lacZ* vector, Em^R^, Ap^R^	Laboratory collection
pHT1618	*E. coli*-*Bt* shuttle, Ap^R^, Tet^R^	[25]
pET21b-gabR	pET-21b containing *gabR* gene, Ap^R^	This study
pHT-PgabT	pHT304-18Z carrying promoter upstream from *gabT*	[8]
pHT-PgabR	pHT304-18Z carrying promoter upstream from *gabR*	[8]
pHT-PgabTDR	pHT304-18Z carrying promoter upstream from *gabT* without GabR binding site	This study
pHT-PgabTDI	pHT304-18Z carrying promoter upstream from *gabT* without the first 21 repeat region of GabR binding site	This study
pHT-PsigL	pHT304-18Z carrying promoter upstream from *sigL*	This study
pHT1618-gabR	pHT1618 containing *gabR* promter and *gabR* gene, Ap^R^	This study
pHT1618-DPAS	pHT1618 containing *gabR* promter and PAS domain deletion fragment, Ap^R^	This study

### DNA manipulation techniques

PCR was performed using *Taq* and KOD DNA polymerase (New England BioLabs Ltd., Beijing, China). Amplified fragments were purified using Axygen purification kits (Silicon Valley, CA, USA). Bt chromosomal DNA was extracted with the Puregene kit (Gentra, Minneapolis, MN, USA). Restriction enzymes and T4 DNA ligase (TaKaRa Biotechnology, Dalian, China) were used according to the manufacturer’s instructions. Oligonucleotide primers (Table 2) were synthesized by Sangon (Shanghai, China). *E. coli* plasmid DNA was extracted using the Axygen Plasmid Extraction Kit. All constructs were confirmed by DNA sequencing (BGI, Beijing, China).

**Table 2:** **Primers used in this study**

**oligonucleotides**	**sequence (5’ - 3’)** ^**a**^
PgabT-F	GATTGCTATGCAATTGGGGTGC
PgabT-R	CCTTTTCTTTACATTGTTTTCTC
gabTRACE	CGAGCGATCTTCACCGCGTTCTCAACT
UPM	AAGCAGTGGTATCAACGCAGAGTACGCGGG
GabR-F	CG*GGATCC*GATGGTTGCAGAAAAGGAACG
GabR-R	GC*GTCGAC*TCCAATGTTTTCTTCCTCCTCTATG
PgabTDR-F	AA*CTGCAG*GAACAAGCCTTGATGTAGCGAA
PgabTDR-1	CTATAAAGACTCTTAAAAAGAAAGTTGGCA
PgabTDR-2	TGCCAACTTTCTTTTTAAGAGTCTTTATAG
PgabTDR-R	CG*GGATCC*CCTTTTCTTTACATTGTTTTCTC
PgabTDI-1	GTCTAATGTGTAAGG TTAATGAGTCTTTAT
PgabTDI-2	ATAAAGACTCATTAACCTTACACATTAGAC
PsigL-F	CCC*AAGCTT*ATAGAGCGGTGGTTTCCGGTAC
PsigL-R	CG*GGATCC*AATAATCTCCCCCTTGTTTCTATTG
PgabRD-F	AA*CTGCAG*GTGCTGGTGTACCGATAAG
PgabRD-R	CG*GGATCC*CGATCCACTTCACTACCTT
CgabR-1	CG*GGATCC*GTGCTGGTGTACCGATAAGT
CgabR-2	CCG*GAATTC*TTATCCAATGTTTTCTTCCTCC
DPAS-F	GGGATCGTTGTGCGTAATCAGCTAAAAAC
DPAS-R	GTTTTTAGCTGATTACGCACAACGATCC

^**a**^Restriction enzyme sites are italics.

### Total RNA isolation and 5′-RACE analysis

For total RNA purification, strain HD73 was grown as previously described in SSM medium until the T7 stage of stationary phase (corresponding to 7 h after the end of the exponential phase) [21]. cDNA synthesis and transcriptional start sites (TSSs) of the *gabT* gene were determined using the SMARTer™ RACE cDNA Amplification Kit (Clontech, Mountain View, CA, USA) according to the manufacturer’s instructions. Gene-specific primers, gabTRACE, and the universal primer mix (UPM) (Table 2) were used to amplify the 5′ end of *gabT* mRNA.

### Expression and purification of GabR protein

The expression plasmid pETgabR containing *gabR* from Bt strain HD73 was constructed by cloning the amplified *gabR* gene, using primers GabR-F (with a 5’ *Bam*HI restriction site) and GabR-R (with a 5’ *Sal*I restriction site), into pET21b previously digested with *Bam*HI and *Sal*I. pETgabR was transferred into *E. coli* BL21(DE3). Transformant *E. coli* cells were grown to exponential phase in LB medium supplemented with ampicillin at 37°C. The expression of GabR-His protein was induced with 1 mM (final concentration) isopropyl-β-D-thiogalactopyranoside for 3 h at 37°C. Cells were harvested by centrifugation at 13,500 × g for 10 min and suspended in 10 mM imidazole NPB buffer (10 mM imidazole, 1 M NaCl, 20 mM sodium phosphate, pH 7.4). Bacteria were lysed on ice by sonication using an ultrasonic cell disruption system (Mini-Beadbeater-96, Biospec). The bacterial lysate was centrifuged at 16,000 × g for 10 min at 4°C, and supernatant containing solubilized GabR-His protein was recovered, filtered through a 0.45-μm membrane filter, and loaded onto a HiTrap chelating column (1 ml, Pharmacia). After binding the protein, the column was washed with 10 mM imidazole NPB solution and the target GabR-His protein was eluted with NPB solution containing a stepwise gradient of imidazole from 100 to 500 mM.

### Gel mobility shift assays

The P*gabT* DNA fragment was obtained by PCR of strain HD73 genomic DNA using primers PgabT-F and PgabT-R, which were previously labeled according to a fluorescent 5′-end 6-FAM modification and confirmed by DNA sequencing. Electrophoresis mobility shift assays (EMSA) were performed as previously described [22] to analyze the binding of purified GabR protein to P*gabT* DNA. Briefly, the P*gabT* DNA probe (0.1 μg) was incubated with different concentrations of purified GabR at 25°C for 20 min in binding buffer (10 mM Tris–HCl, 0.5 mM dithiothreitol (DTT), 50 mM NaCl, 500 ng poly (dI:dC), pH 7.5 and 4% (v/v) glycerol) in a total volume of 20 μl. The DNA-protein mixtures were applied to non-denaturing 5% (w/v) polyacrylamide gels in TBE buffer (90 mM Tris-base, 90 mM boric acid, 2 mM EDTA, pH 8.0) for resolution of the complexes, using a Mini-PROTEAN system (Bio-Rad) at 160 V for 1 h. Signals were visualized directly from the gel with the FLA Imager FLA-5100 (Fujifilm). The specificity of the shift was confirmed using poly (dI:dC), and GST protein, bovine serum albumin (BSA), and as negative controls, the *cry1Ac* and *gerE* promoters (which do not bind to GabR protein; data not shown). Increasing concentrations (0.1-50 μM) of SSA or GABA were incubated with the P*gabT* probe and GabR to analyze the effect of SSA and GABA on the binding of GabR and P*gabT*.

### DNase I footprinting assays

DNase I footprinting assays were performed based on a fluorescent labeling procedure [23]. Briefly, *gabT* promoter DNA was PCR-amplified using the fluorescently-labeled primers PgabT-F and PgabT-R (Table 2) and purified from an agarose gel. The labeled P*gabT* DNA probe (120 ng) was incubated for 20 min at 25°C with the indicated concentrations of purified GabR and BSA in a total volume of 50 μl of binding buffer (described above for EMSA). DNase I digestion was then carried out for 1 min at 25°C and stopped with stop buffer (Promega). After phenol-chloroform extraction and ethanol precipitation, the samples were loaded on an Applied Biosystems 3730 DNA genetic analyzer together with an internal-lane size standard (ROX-500, Applied Biosystems). A dye primer-based sequencing kit (Thermo) was used to precisely determine the sequences after their alignment wtih the capillary electrophoresis results from the reactions. Electropherograms were analyzed with GeneMarker v1.8 (Applied Biosystems).

### Construction of the P*gabT-lacZ* fusion bearing a deletion of GabR-binding site

A fragment containing the *gabT* promoter with the GabR-binding site deleted was cloned in fusion with the *lacZ* gene. The construct was obtained as follows: The downstream and upstream regions of the *gabT* promoter were amplified in two PCR reactions, using strain HD73 DNA as the template, and PgabTDR-F/PgabTDR-2 and PgabTDR-1/PgabTDR-R primers, respectively. These DNA fragments were fused by overlapping PCR using the primer pair PgabTDR-F/PgabTDR-R. The resulting PCR products were digested with *Pst*I and *Bam*HI, purified, and ligated into the low-copy-number vector pHT304-18Z [24], which harbors a promoterless *lacZ* gene. The recombinant plasmid named pHT-P*gabTDR*, which has a deletion of 60 bp corresponding to the GabR-binding site, was introduced into Bt strain HD73, yielding the transformant strain HD(P*gabTDR-lacZ*). Another recombinant plasmid, pHT-P*gabTDI*, which has a deletion of the first 21-bp repeat located in the GabR-binding site region, was constructed by a similar method. The downstream and upstream regions of the *gabT* promoter were amplified in two PCR reactions, using strain HD73 DNA as the template, and PgabTDR-F/PgabTDI-2 and PgabTDI-1/PgabTDI-R primers, respectively. These DNA fragments were fused by overlapping PCR using the primer pair PgabTDR-F/PgabTDR-R. The PCR products were digested with *Pst*I and *Bam*HI, purified, and ligated into the vector pHT304-18Z. The resulting plasmid, pHT-P*gabTDI*, was introduced into Bt strain HD73 yielding the transformant strain HD(P*gabTDI-lacZ*).

### Construction of *sigL* and *gabR* promoters with *lacZ* gene fusion

In strain HD73, Sigma 54 is encoded by the *sigL* gene. 313 bp fragment from the *sigL* promoter was amplified from strain HD73 DNA using primers PsigL-F (with 5′- *Hin*dIII) and PsigL-R (with 5′-*Bam*HI) (Table 2). The *Hin*dIII-*Bam*HI restriction fragment of the *sigL* promoter was cloned into the vector pHT304-18Z. The recombinant plasmid, pHT-P*sigL*, was introduced into Bt strain HD73 yielding the transformant strain HD (P*sigL-lacZ*).

A fragment containing 633 bp of the *gabR* promoter was previously cloned [8]. Here, a truncated *gabR* promoter fragment of 580 bp was constructed, deleting the −12/-24 conserved element. The 580-bp *gabR* promoter fragment was amplified from strain HD73 DNA using PgabRD-F (with 5′- *Pst*I) and PgabRD-R (with 5′-*Bam*HI) primers (Table 2). The *Pst*I-*Bam*HI fragment of *gabR* promoter was then ligated into the vector pHT304-18Z [24]. The recombinant plasmid pHT-P*gabRD* was introduced into Bt strain HD73, and the resulting strain was named HD(P*gabRD-lacZ*).

### Complementation of the *gabR* mutant and deletion of PAS domain

A DNA fragment containing *gabR* and the *gabR* promoter was amplified with CgabR-1 and CgabR-2 primers (Table 2) using Bt strain HD73 DNA as template. The PCR product (1,974 bp) was digested with *Bam*HI and *Eco*RI and ligated into plasmid pHT1618 [25]. The resulting plasmid (pHT1618-gabR) was amplified in *E. coli* and introduced into the Bt mutant strain Δ*gabR*(P*gabT*-*lacZ*). This plasmid complements the *gabR* mutant strain and allows evaluation of the expression of the *gabT* promoter-*lacZ* fusion.

A similar plasmid carrying the *gabR* promoter and *gabR* gene but without the PAS domain encoded sequence, was constructed by amplifiying two DNA fragments using strain HD73 DNA as the template and CgabR-1/DPAS-R and DPAS-F/CgabR-2 primers, respectively (Table 2). The amplified fragments were fused by an overlapping PCR using CgabR-1 and CgabR-2 primers. The final PCR product (1,689 bp) was digested with *Eco*RI and *Bam*HI, purified and ligated into plasmid pHT1618. The resulting plasmid (pHT1618-DPAS) was amplified in *E. coli* and introduced into the Bt mutant strain Δ*gabR*(P*gabT*-*lacZ*). This plasmid complements the *gabR* strain, with a *gabR* gene where the PAS domain (amino acids 33–128 of GabR) was deleted, and allows evaluation of the *gabT* promoter-*lacZ* fusion in β-galactoside activity assays. The resulting strains C*gabR*(P*gabT*) and DPAS(P*gabT*) were selected based on their erythromycin and tetracycline resistance and identified by PCR.

### β-galactosidase assays

Bt strains containing *lacZ* transcriptional fusions were cultured in AOAC medium (supplemented with 7 mM glucose) at 30°C and 220 rpm. GABA or SSA at concentrations of 0, 0.2, 1 and 5 mM was added to the cultures when the OD_600_ reached 1.0 (mid-exponential phase, defined as A_0_). For P*gabT* and P*gabR* activity assay without GABA or SSA, T_0_ is the end of the exponential phase (OD_600_ = 2.0-2.2), and Tn is n hours after the end of the exponential phase. Samples of 2.0 ml were taken at 1-h intervals after induction. Cells were harvested by centrifugation for 1 min at 12,000 × g, and the supernatant was discarded. The β-galactosidase-specific activities were determined as previously described [26] and expressed as Miller units. The values reported represent averages from at least three independent assays.

## Results

### Determination of the transcriptional start site of *gabT*

**Figure 1:**
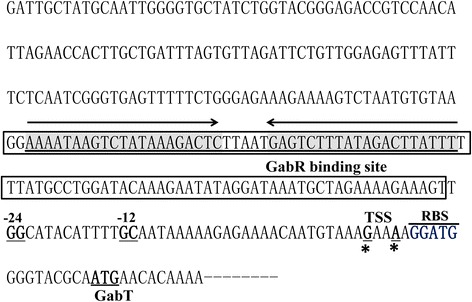
**Nucleotide sequence of the** ***gabT*** **upstream region.** Transcriptional start sites (TSSs, indicated by asterisks) are located 16 and 19 bp upstream from the start codon of the *gabT* gene (underlined). The putative ribosome-binding-site (RBS) is indicated by an upper line. The −12, −24 sequences are double underlined. The GabR binding site (boxed region) maps 58 bp upstream of the start codon of *gabT*. A 21-bp repeat region (underlined, gray and arrow) maps 105 bp upstream of the *gabT* start codon.

To determine the transcription start site (TSS) of *gabT*, 5′-RACE analysis was performed as described in the Materials and Methods. According to the sequences of 16 random clones, a G located 19 bp upstream from the start codon was identified in eight, and an A residue located 16 bp upstream of the start codon in the remaining eight. Thus two TSSs were located 16 and 19 bp upstream of the ATG start codon of *gabT* (Figure 1). The typical ribosome binding site (RBS) sequence (−AGGATG−) was identified at an appropriate distance upstream from the start codon of *gabT*. The sequence TGGCATACATTTGCA was identified upstream of the *gabT* start codon and was similar to the −12/−24 consensus sequence (BY*GG*CMYRNNNYY*GC*W) of Sigma 54-binding sites [27].

However, the two TSSs were located 25 bp and 28 bp from the putative −12 promoter element. In several Sigma 54-dependent promoters, the initiation of transcription is not precisely located 12 nt downstream from the conserved GC element [28]. For instance, in the case of the *Rhodospirillum centenum cheAY* promoter, the TSS is located 8 nt downstream of the −12 element [29]. In the *Pseudomonas aeruginosa algD* promoter, the TSS and −12 elements are 21 nt apart [30].

### Identification of a GabR-binding site in the *gabT* promoter fragment

**Figure 2:**
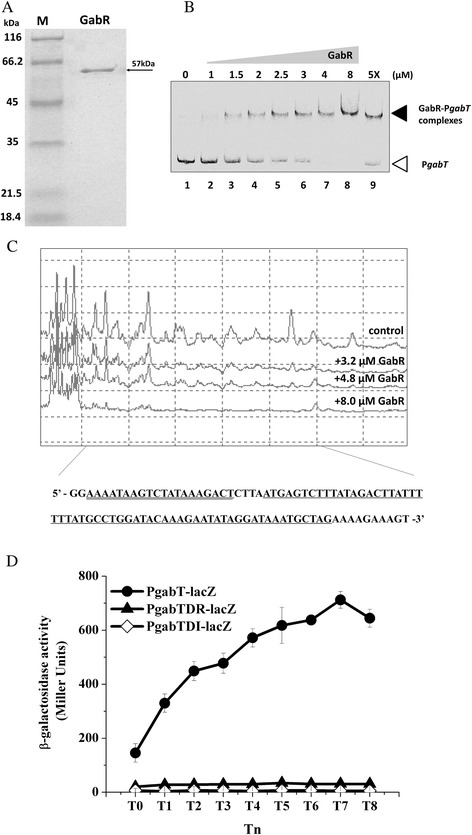
**Identification of the GabR-binding site in the** ***gabT*** **promoter.** Panel **A**, SDS-PAGE analysis of GabR (57 kDa) expressed in *E. coli* (pET21b-gabR) and purified by nickel affinity column chromatography. M, protein marker. Panel **B**, Electrophoresis mobility shift assay (EMSA) of the *gabT* promoter fragment (286 bp) after interaction with GabR. Lane 1, FAM-labeled P*gabT* probe; lanes 2–8, incubation of the probe with increasing concentrations of purified GabR indicated at the top of the figure. Each lane contained 0.1 μg of probe. Lane 9, incubation of a five-fold greater amount of unlabeled P*gabT* probe mixed with labeled P*gabT* probe and 8 μM GabR. Panel **C**, protection of a 96-bp sequence in the *gabT* promoter by GabR, as revealed in a DNase I footprinting protection assay. The fluorograms correspond to the control DNA (10 μM BSA) and to the protection reactions (with 3.2, 4.8 and 8 μM of GabR). Panel **D**, β-galactosidase activity assay of the *gabT* promoter with the GabR-binding site (●), without the 60-bp GabR-binding site (▲), and without the first 21-bp repeat region of the GabR-binding site (◊). The deletion sequence is that underlined in Figure 2C. T_0_ is the end of exponential phase, and Tn is n hours after T_0_.

To determine whether GabR is able to bind the *gabT* promoter, GabR-His protein was expressed in *E. coli* and purified to near-homogeneity using Ni^2+^-affinity chromatography. The mobility of GabR-His in SDS-PAGE corresponded well to its predicted molecular mass of 57 kDa (Figure 2A). The ability of GabR to bind to a DNA fragment containing the P*gabT* (286 bp) promoter was examined by EMSA. FAM-labeled fragments containing the promoter regions of *gabT* were incubated with different amounts of GabR and assayed for the formation of protein-DNA complexes. Slower-migrating probe-protein complexes were observed upon incubation with increasing amounts of GabR (Figure 2B). A five-fold molar excess of unlabeled P*gabT* probe competed with the labeled P*gabT* probe, indicating specific binding (Figure 2B, lane 9). GabR did not bind to other labeled promoters from strain HD73, such as *cry1Ac* and *gerE* (data not shown). These results indicate that GabR recognizes and specifically binds to sequences within the *gabT* promoter fragment.

To precisely determine the GabR-binding site in the *gabT* promoter, DNase I footprinting assays were carried out using the same *gabT* promoter fragment used in the EMSA. A 96-bp fragment was protected by GabR binding (Figure 2C) (corresponding to the boxed sequence in the *gabT* region shown in Figure 1). Moreover, a 21-bp repeat region mapped 105 bp upstream of the start codon of *gabT* (Figure 1), with the 21-pb inverted repeat separated by 5 bp (shaded gray in Figure 1).

To determine whether the proposed 96-bp sequence is the GabR binding site *in vivo*, a 60-bp fragment of the binding site was deleted from the *gabT* promoter and the promoter carrying the deletion was fused to *lacZ* (P*gabTDR-lacZ*). The deleted sequence is underlined in Figure 2C. Another deletion fragment, in which the first 21-bp repeat located at the GabR-binding site region was removed, was similarly fused to *lacZ* (P*gabTDI-lacZ*). The deleted sequence is double-underlined in Figure 2C. The β-galactosidase assay showed that the activities of HD(P*gabTDR-lacZ*) and HD(P*gabTDI-lacZ*) strains were sharply decreased compared with that of wild-type strain HD73 carrying the *gabT* promoter fused to *lacZ* (P*gabT-lacZ*) (Figure 2D). This result shows that disruption of the proposed GabR-binding site prevents GabT expression *in vivo*.

### GABA and SSA induce *gabT* expression

**Figure 3:**
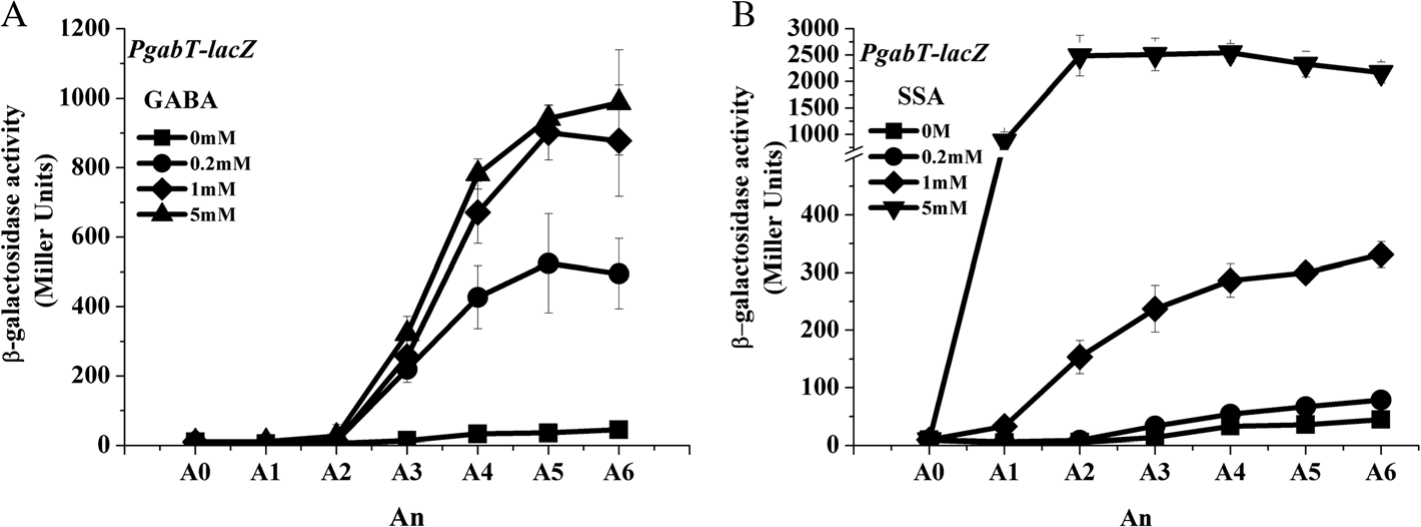
**Induction of** ***gabT*** **transcription by GABA and SSA.** Bt strains containing a P*gabT-lacZ* transcriptional fusion were cultured in AOAC medium. At OD_600_ = 1.0 (A_0_), GABA and SSA were added at different concentrations. Samples were collected at 1-h intervals for 6 h after induction and β-galactosidase activity was assayed. Panel **A**, GABA was added to strain HD(P*gabT*-*lacZ*) at a final concentration of 0 mM (■), 0.2 mM (●), 1 mM (◆) and 5 mM (▲). Panel **B**, SSA was added to strain HD(P*gabT*-*lacZ*) at a final concentration of 0 mM (■), 0.2 mM (●), 1 mM (◆) and 5 mM (▼). A_0_ is mid-exponential phase (OD_600_ = 1.0), and An is n hours after A_0_.

The P*gabT-lacZ* fusion showed lower activity in AOAC medium (Figure 3) than in SSM (Figure 2D). To analyze the inducible activity of the *gabT* promoter, cells were cultured in AOAC medium. The addition of 0.2, 1 and 5 mM GABA or SSA induced the expression of a P*gabT-lacZ* fusion (Figure 3A and B), confirming the induction of *gabT* expression by these two signaling molecules. However, they differed in their *gabT* transcription induction patterns: GABA caused induction after A_3_ (3 hours after the mid-exponential phase), whereas SSA caused nearly immediate induction. These results might be explained if we hypothesize that SSA is the direct signal, while GABA is the precursor of SSA signal synthesis.

### Induction of *gabT* promoter activity by GABA and SSA depends on Sigma 54 and GabR

**Figure 4:**
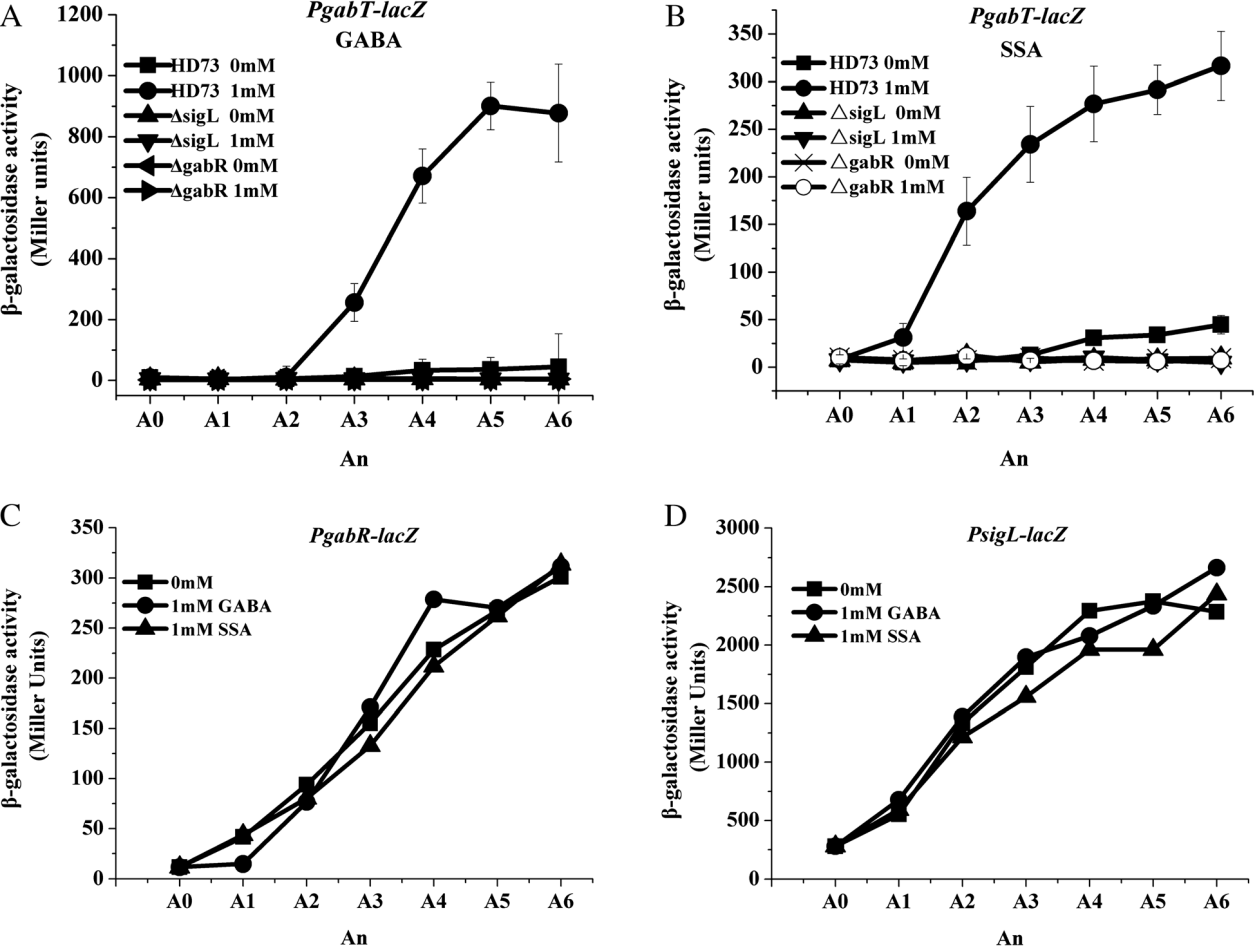
**Analysis of the transcriptional activation of** ***gabT, gabR*** **and** ***sigL*** **promoters.** Panel **A**, analysis of β-galactosidase activity induced by the *gabT* promoter in strain HD73, the *sigL* mutant and the *gabR* mutant with or without GABA. Strain HD(P*gabT*-*lacZ*) without (■) and with (●) 1 mM GABA, strain Δ*sigL*(P*gabT*-*lacZ*) without (▲) and with (▼) 1 mM GABA, strain Δ*gabR*(P*gabT*-*lacZ*) without (◀) and with (▶) 1 mM GABA. Panel **B**, analysis of β-galactosidase activity induced by the *gabT* promoter in strain HD73, the *sigL* mutant and the *gabR* mutant with or without SSA. Strain HD(P*gabT*-*lacZ*) without (■) and with (●) 1 mM SSA, strain Δ*sigL*(P*gabT*-*lacZ*) without (▲) and with (▼) 1 mM SSA, strain Δ*gabR*(P*gabT*-*lacZ*) without (×) and with (○) 1 mM SSA. Panel **C**, analysis of β-galactosidase activity induced by the *gabR* promoter in strain HD73 with or without GABA and SSA. Strain HD(P*gabR*-*lacZ*) without GABA or SSA (■), with 1 mM GABA (●), and with 1 mM SSA (▲). Panel **D**, analysis of β-galactosidase activity induced by the *sigL* promoter in strain HD73 with or without GABA and SSA. Strain HD(P*sigL*-*lacZ*) without GABA or SSA (■), with 1 mM GABA (●), and with 1 mM SSA (▲). A_0_ is mid-exponential phase (OD_600_ = 1.0), and An is n hours after A_0_.

In a previous study, we demonstrated that *gabT* expression is controlled by Sigma 54, via the transcription activator GabR [8]. Here, we analyzed the effect of 1 mM GABA or SSA on P*gabT* transcription activity in *sigL* (Sigma 54 coding gene in Bt) or *gabR* mutants. Neither GABA nor SSA induced activation of the *gabT* promoter in Δ*sigL* or Δ*gabR* (Figure 4A and B). These data showed that GabR and Sigma 54 are absolutely necessary for *gabT* transcription and that GABA- or SSA-induced *gabT* transcription is controlled by Sigma 54 and activated by GabR bEBP.

### P*gabT* induction is not caused by GABA or SSA enhancement of the transcriptional activity of *gabR* or *sigL*

To determine whether GABA and SSA induced transcription of the *gabT* promoter by affecting *sigL* and *gabR* transcription, strains carrying *sigL* and *gabR* promoter fusions with *lacZ* were used [HD(P*sigL-lacZ*) and HD(P*gabR-lacZ*)]. The addition of GABA and SSA had no effect on either P*gabR* or P*sigL* transcriptional activities (Figure 4C and D), suggesting that the GABA- and SSA-induced transcription from the *gabT* promoter is not due to the increased expression of *sigL* or *gabR*. Increasing concentrations of GABA and SSA did not enhance the binding of GabR to the *gabT* promoter (Additional file 1: Figure S1). Thus, the induction of P*gabT* by GABA and SSA is not caused by activating GabR and DNA binding.

### The *gabR* promoter is not a Sigma 54-dependent promoter

**Figure 5:**
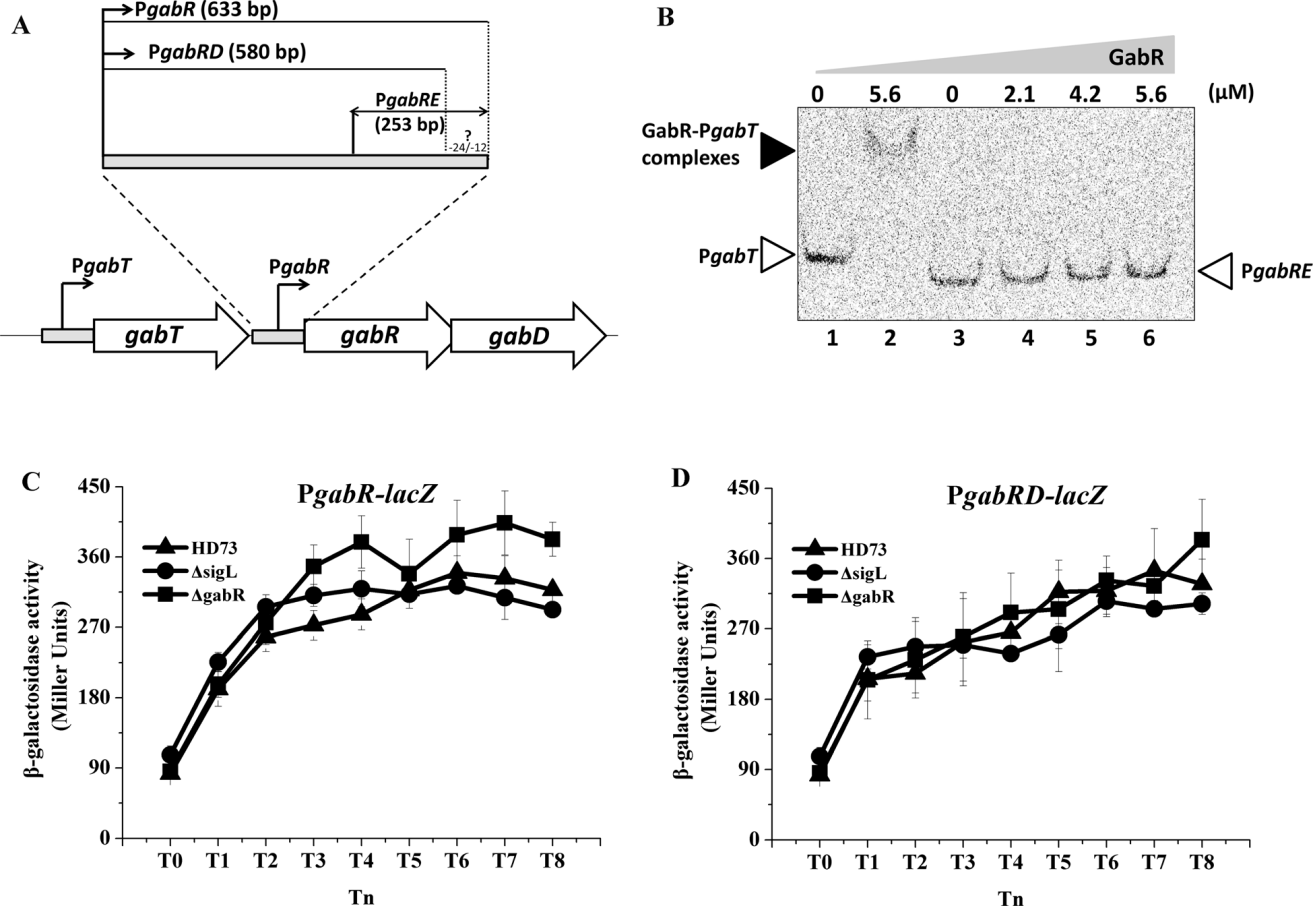
**Analysis of the role of Sigma 54 and GabR on** ***gabR*** **transcription.** Panel **A**, Model of the *gabR* upstream region. The P*gabR* (633 bp) fragment contains the wild-type promoter, the P*gabRD*(580 bp) fragment of the *gabR* promoter without the −12/−24-like elements, and the P*gabRE*(253 bp) fragment is used for GabR binding. Panel **B**, EMSA for P*gabRE* after interaction with GabR. Lane 1, FAM-labeled P*gabT* probe; Lane 2, probe P*gabT* was incubated with 5.6 μM of purified GabR. Lane 3–6, the FAM-labeled P*gabRE* probe was incubated with increasing concentrations of purified GabR as shown at the top of the figure. Each lane contained 0.1 μg of probe. **C**. β-galactosidase activity assay of P*gabR* in wild-type strain HD73 (▲), the *sigL* mutant (●) and the *gabR* mutant (■). **D**. β-galactosidase activity assay of P*gabRD* in wild-type strain HD73 (▲), the *sigL* mutant (●) and the *gabR* mutant (■). T_0_ is the end of exponential phase, and Tn is n hours after T_0_.

In the following, we provide additional evidence that transcription from the *gabR* promoter is not dependent on SigL, nor is it activated by GabR, which differs from the conclusion reached in our previous study [8]. EMSA performed to analyze the binding of GabR to the P*gabR* promoter showed that GabR did not bind to its own *gabR* promoter (Figure 5B). The β-galactosidase activity of the *gabR* promoter was tested again in the *sigL* and *gabR* mutants. The results failed to showed significant difference in the *gabR* promoter activities of wild-type strain HD73 and the *sigL* and *gabR* mutants (Figure 5C). Furthermore, deletion of a Sigma 54-like −12/−24 conserved region in the *gabR* promoter (P*gabRD* highlighted in Figure 5A) had no effect on P*gabR* transcription (Figure 5D). It should be noted that several reports have shown that deletion of one or more nucleotides in the stretch included in the −24/−12 elements of Sigma 54-dependent promoters abolishes promoter function [31,32]. Those studies proposed that there is a stringent requirement for these motifs to be positioned on the same face of the DNA helix as a necessary condition for the binding of RNA polymerase and Sigma 54 [28]. Since there are only 11 nt in −24/−12 elements of the *gabR* promoter, it is not a typical Sigma 54-dependent promoter. Furthermore, GABA and SSA had no effect on the binding of GabR to the *gabT* promoter, as judged by EMSA assays (data not shown), suggesting that GABA and SSA activation of P*gabT* is not caused by enhanced GabR-binding to DNA.

### The PAS domain of GabR plays a role in the GABA-inducible transcription of *gabT*

**Figure 6:**
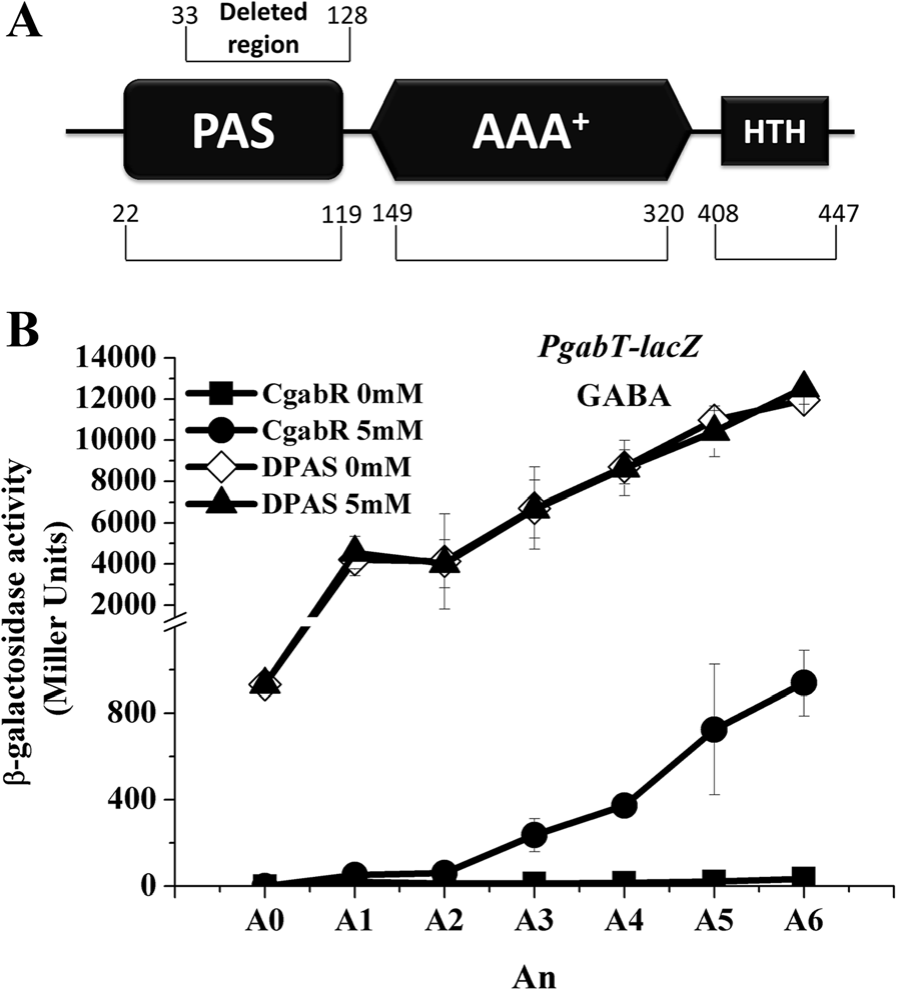
**Analysis of the role of the PAS domain of GabR in** ***gabT*** **transcription.** Panel **A**, Domain organization of GabR. GabR is a Sigma 54-dependent activator that has three typical domains, the N-terminal regulatory domain (PAS domain, amino acids 22–119), the central AAA^+^ domain (amino acids 149–320) and C-terminal DNA-binding domain (HTH, amino acids 408–447). In the PAS domain deletion strain DPAS, the PAS domain from amino acids 33 to 128 is deleted from GabR. The *gabR* promoter region (606 bp) was used to construct the pHT1618-gabR and pHT1618-DPAS plasmids. Panel **B**, analysis of β-galactosidase activity induced from the *gabT* promoter in the Bt strain complemented with GabR or with the PAS deletion mutant in the presence or absence of GABA. Strain CgabR without (■), and with (●) 5 mM GABA, strain DPAS without (◊), and with (▲) 5 mM GABA. A_0_ is mid-exponential phase (OD_600_ = 1.0), and An is n hours after A_0_.

GabR is a Sigma 54-dependent transcriptional activator containing three typical domains, similar to those in other bEBPs, including a PAS domain in the N-terminal region (Figure 6A). To determine the role of PAS domain in GABA-induced transcription of *gabT*, a PAS domain deletion in GabR was constructed and its capacity to induce the *gabT* promoter was compared with that of wild-type GabR. The GabR ΔPAS mutant (DPAS) and GabR wild-type (CgabR) proteins were then used to complement a GabR mutant. The transcriptional activity of *gabT* was determined by measuring β-galactosidase activity of the fusion protein (see Methods). As shown in Figure 6B, *gabT* promoter activity in strain DPAS(P*gabT*) was higher than that of strain C*gabR*(P*gabT*) without the addition of GABA. Interestingly, GABA had no effect on the transcriptional activity of DPAS (PgabT) (Figure 6B). These data suggest that the PAS domain in GabR is repressing its enhancer transcriptional activity on the *gabT* promoter. Repression is released upon GABA addition, whereupon transcription is induced.

## Discussion

The structure of the *gab* gene cluster in Bt is different from those found in *E. coli* and *B. subtilis* [8]. We confirmed that expression of *gabT* in Bt is positively regulated by GabR, which is a Sigma 54-dependent transcriptional activator, and that GabR specifically binds to the *gabT* promoter. Transcriptional activation of *gabT* by GabR is mediated by the signal molecules GABA and SSA. In Bt, GabR is not involved in *gabR* transcriptional activation and GABA and SSA had no effect on *sigL* or *gabR* transcription. By contrast, in *B. subtilis*, *gabD* and *gabT* form a GABA-inducible operon, with GabR, encoded by a divergent gene, *gabR*, acting as a transcriptional regulator of both the *gabTD* operon and its own gene [33]. In *B. subtilis*, the presence of both GABA and pyridoxal 5′-phosphate (PLP) is required for GabR-dependent activation of the *gabT* promoter. The efficiency of the GabR-dependent repression of the *gabR* transcript is not affected by the addition of PLP or GABA [33]. In this work we showed that the GABA-inducible mechanism in Bt is different from that in *B. subtilis*.

These different induction mechanisms can be attributed to the different GabR structures found in Bt and *B. subtilis*. GabR of *B. subtilis* is a member of the understudied MocR/GabR subfamily of the GntR family of transcription regulators [34]. GabR in *B. subtilis* has a short N-terminal HTH-containing domain and a long C-terminal domain that is similar to full-length aminotransferases or to the PLP-binding domain of aminotransferases [33]. The GabR structure provides a model for DNA binding at the *gabR-gabT* locus by one or more GabR homodimers. GabR aminotransferase-like activity involving GABA and PLP is not essential to its primary function as a transcriptional regulator [34]. However, GabR in Bt is a Sigma 54-dependent transcriptional activator, also referred to as a bacterial enhancer binding protein (bEBP). GabR comprises three domains (Figure 6A), including an N-terminal PAS domain. Here, we showed that the PAS domain of GabR, which is the signal sensor domain, is required for the regulation of GABA-inducible transcriptional activity of the *gabT* promoter.

The role of the N-terminal regulatory domain of bEBPs has been documented in many Sigma 54-dependent transcriptional activators, whereas little is known about the function of the PAS domain, even though PAS domain is present in many bEBPs. Indeed, only the PAS domain of the bEBP-like protein TyrR has been investigated [16]. TyrR contains both a PAS domain and an ACT domain and it facilitates the activation or repression of the transcription of genes involved in aromatic amino acid biosynthesis and transport. In this study, we found that deletion of the PAS domain of GabR in Bt results in induced *gabT* expression and that induction is not dependent on GABA. The *gabT* promoter activity in the PAS domain deletion strain was higher than that in the strain containing wild-type GabR. These data suggests that the PAS domain of GabR acts as a signal sensor domain that is repressing GabR enhancer transcriptional activity on the *gabT* promoter. Repression is released upon GABA addition, whereupon transcription is induced. This model agrees with the proposed function of the N-terminal regulatory domain of bEBPs in modulating the activity of bEBPs. PAS domains have been shown to bind a diverse array of ligands [15]. In the case of Bt, it is proposed that the PAS domain of GabR binds to GABA and/or SSA. It will be interesting to determine whether the PAS domain of other bEBPs functions in a similar way, i.e., by repressing bEBPs enhancer transcriptional activity after the sensing of signal ligands.

## Conclusions

**Figure 7:**
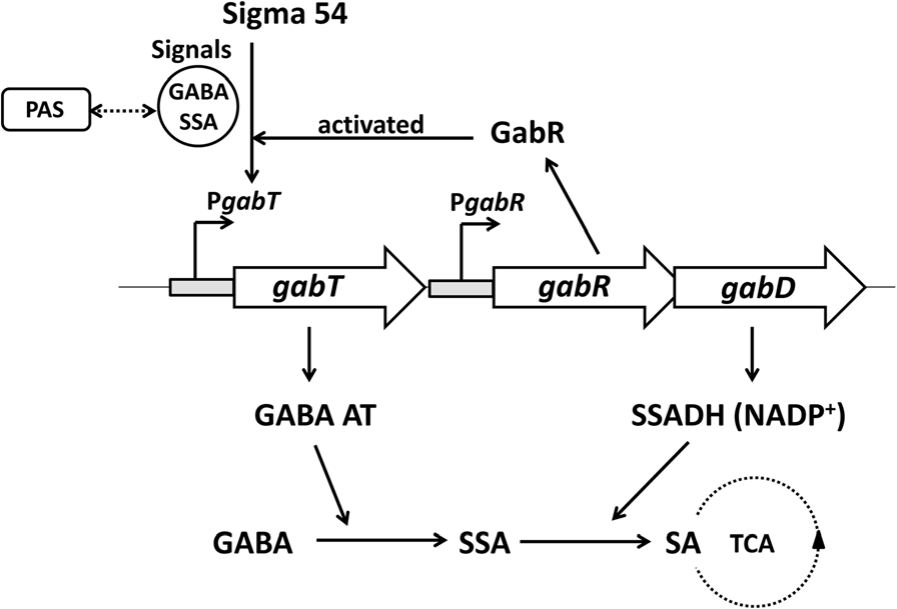
**Schematic diagram of the regulatory pattern of the** ***gab*** **gene cluster in Bt.** The hollow arrows (from left to right) indicate the *gabT*, *gabR*, and *gabD* genes. P*gabT* and P*gabR* are the putative promoter regions of *gabT* and *gabR*, respectively. The dashed circle indicates the TCA cycle. There are two transcriptional units, *gabT* and *gabRD*, in the *gab* gene cluster involved in the GABA shunt. GABA and SSA are the signals that induce *gabT* transcription via the PAS domain of GabR. Transcription of *gabT* is controlled by Sigma 54, and activated by GabR, via the binding to the 96-bp fragment of the *gabT* promoter.

The data presented here improve our understanding of the regulation of the *gab* gene cluster in Bt and form the basis of a new regulatory model is proposed herein (Figure 7). Two transcriptional units, *gabT* and *gabRD*, in the *gab* gene cluster are involved in the GABA shunt. GABA and SSA are the signals involved in *gabT* transcription, via the PAS domain of GabR. Transcription of *gabT* is controlled by Sigma 54, and activated by GabR, through binding to a 96 bp region on the *gabT* promoter. The PAS domain of GabR acts as a signal sensor domain, modulating GabR activity. The data suggest that the PAS domain in GabR is repressing its enhancer transcriptional activity on the *gabT* promoter. Repression is released upon GABA addition, whereupon transcription is induced.

## Additional file

Additional file 1: Figure S1. EMSA for GabR-P*gabT* with different concentration of SSA (A) or GABA (B). Lane 1, FAM-labeled P*gabT* probe; Lane 2-6, Incubation of P*gabT* probe and 2.5μM GabR with the increasing concentrationsof SSA or GABA indicated at the top of the figure. Each lane contained 0.1 μg of probe.
