# Supplementary material for: Activation of gab cluster transcription in Bacillus thuringiensis by γ-aminobutyric acid or succinic semialdehyde is mediated by the Sigma 54-dependent transcriptional activator GabR
Source: BMC Microbiol. 2014 Dec 20;14:2317. doi: 10.1186/s12866-014-0306-3 (PMC4279683; doi:10.1186/s12866-014-0306-3)
Supplement: Additional file 1: Figure S1. — EMSA for GabR-PgabT with different concentration of SSA (A) or GABA (B). Lane 1, FAM-labeled PgabT probe; Lane 2-6, Incubation of PgabT probe and 2.5μM GabR with the increasing concentrationsof SSA or GABA indicated at the top of the figure. Each lane contained 0.1 μg of probe. [file 12866_2014_306_MOESM1_ESM.pdf]

Figure S1

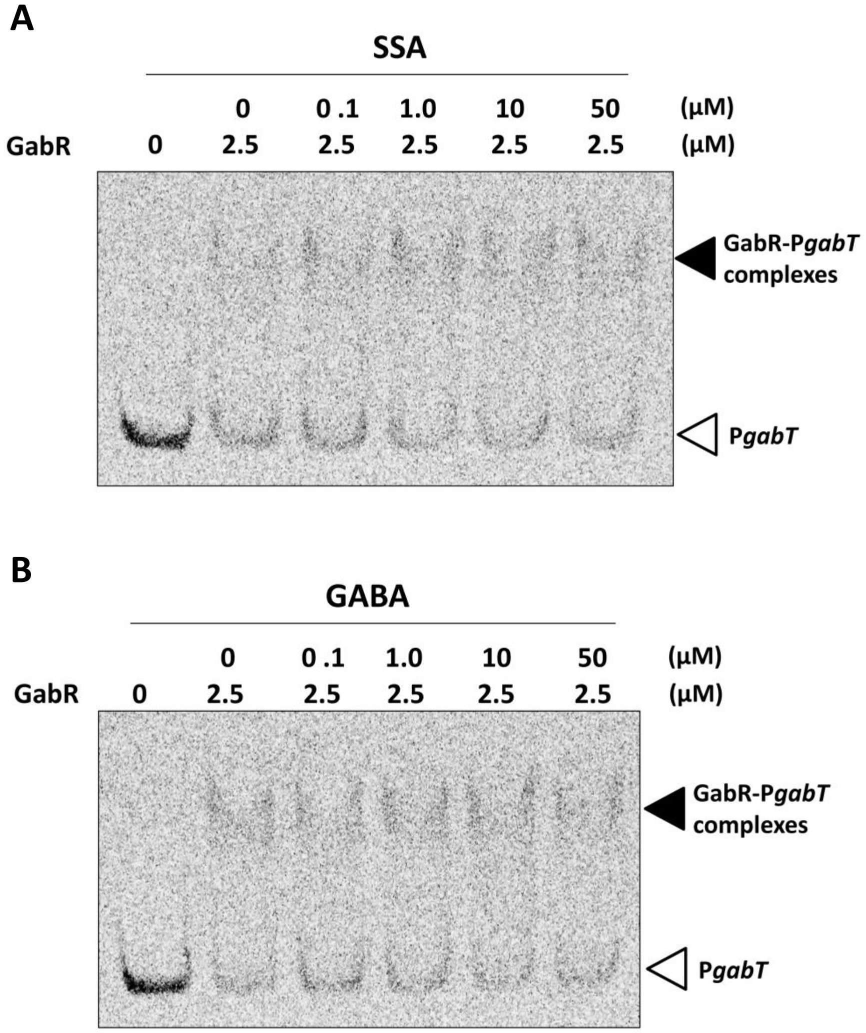

Figure S1. EMSA for GabR-P*gabT* with different concentration of SSA (A) or GABA (B). Lane 1, FAM-labeled P*gabT* probe; Lane 2-6, Incubation of P*gabT* probe and 2.5 μM GabR with the increasing concentrations of SSA or GABA indicated at the top of the figure. Each lane contained 0.1 μg of probe.
